# Identification of autoantibodies against L1CAM in patients with schizophrenia

**DOI:** 10.1016/j.bbih.2026.101288

**Published:** 2026-06-15

**Authors:** Shingo Katayama, Yukiko Motokawa, Gayatri Nayanar, Saori Toyoda, Hiroaki Hori, Yohsuke Yagi, Sayuri Ishiwata, Kinya Ishikawa, Hiroshi Kunugi, Hidehiko Takahashi, Hiroki Shiwaku

**Affiliations:** a Department of Psychiatry and Behavioral Sciences, Institute of Science Tokyo, Yushima, Bunkyo-ku, Tokyo, 113-8510, Japan; b Department of Behavioral Medicine, National Institute of Mental Health, National Center of Neurology and Psychiatry, Tokyo, 187-8553, Japan; c Department of Neurology and Neurological Science, Institute of Science Tokyo, Tokyo, Japan; d Department of Personalized Genomic Medicine for Health, Institute of Science Tokyo, Tokyo, Japan; e Department of Psychiatry, Teikyo University School of Medicine, Tokyo, 173-8605, Japan

**Keywords:** Schizophrenia, L1CAM, anti-L1CAM autoantibody, Autoantibody

## Abstract

Synaptic autoantibodies have been implicated in the pathophysiology of a subset of schizophrenia cases. The identification of novel autoantibodies in schizophrenia may therefore provide insights into previously unrecognized disease mechanisms. Here, we report the presence of autoantibodies against L1 cell adhesion molecule (L1CAM), a membrane protein expressed in neurons that contributes to cell adhesion and synapse formation. Using a cell-based assay, we analyzed 387 patients with schizophrenia and 362 healthy controls. Anti-L1CAM autoantibodies were detected in two patients with schizophrenia but not in healthy controls, and were also identified in the cerebrospinal fluid (CSF). Analysis using L1CAM deletion constructs indicated that the Ig1 domain of L1CAM is a major target of these autoantibodies. Administration of purified IgG from these patients into the CSF of mice was associated with impaired cognitive performance. Patients positive for anti-L1CAM autoantibodies also exhibited marked cognitive deficits. Together, these findings suggest that anti-L1CAM autoantibodies may be associated with disease mechanisms in a small subset of schizophrenia cases.

## 1 Introduction

Autoantibodies targeting neural molecules are known to be associated with autoimmune encephalitis and have been implicated in autoimmune psychosis (Graus et al., 2016; Pollak et al., 2020). Recent studies have suggested that a broad spectrum of neuronal autoantibodies may be associated with psychotic disorders, supporting the concept that autoimmune mechanisms contribute to a subset of psychosis (Hansen, 2023). To date, autoantibodies targeting neuronal surface receptors, including NMDA and GABA receptors, have been identified (Dalmau, 2016; Prüss, 2021). In addition, several other neuronal autoantibodies have been reported in psychotic disorders, including those targeting CASPR2, mGluR5, and glycine receptors (Hansen, 2023).

In this context, the concept of autoimmune psychosis, which initially focused on acute psychosis, has been extended to the hypothesis that autoimmune mechanisms may also contribute to more typical forms of schizophrenia. Consistent with this hypothesis, evidence suggests a role for autoimmunity and autoantibodies in the pathogenesis of schizophrenia (Hansen, 2023; Shiwaku, 2025). Epidemiological studies have associated schizophrenia with autoimmune diseases, and genetic analyses have revealed immune-related risk variants (Benros et al., 2011; Cao et al., 2023; Network and Pathway Analysis Subgroup of the Psychiatric Genomics Consortium, 2015; Schizophrenia Working Group of the Psychiatric Genomics Consortium, 2014; Cullen et al., 2019; Eaton et al., 2006; Wang et al., 2018). Furthermore, autoantibodies against NMDA and GABA receptors, NCAM1, and NRXN1 have been detected in patients with schizophrenia (Lennox et al., 2017; Luykx et al., 2024; Shiwaku, 2025; Shiwaku et al., 2020, 2022, 2023). Importantly, NCAM1 and NRXN1 autoantibodies have been reported to induce cognitive impairment, prepulse inhibition deficits, and impaired social behaviors when administered to mice, suggesting potential pathogenic effects (Shiwaku et al., 2022, 2023). These findings suggest that neuronal autoantibodies may be associated with schizophrenia and could potentially contribute to disease mechanisms. The identification of novel neuronal autoantibodies may also aid in elucidating the underlying biology of schizophrenia.

Several neuronal autoantibodies have been reported to target neural adhesion molecules, including those against NCAM1, NRXN1, and NRXN3, which suggests that neural adhesion molecules may represent potential targets of previously unidentified autoantibodies (Gresa-Arribas et al., 2016; Shiwaku et al., 2022, 2023). NCAM1 is a neural cell adhesion molecule containing immunoglobulin (Ig)-like domains and belongs to the Ig superfamily (Sytnyk et al., 2017). We previously reported that anti-NCAM1 autoantibodies in patients with schizophrenia recognize its Ig domain (Shiwaku et al., 2022).

The Ig superfamily includes numerous neural adhesion molecules expressed at synapses; however, autoantibodies targeting Ig superfamily adhesion molecules have so far been reported only for NCAM1 in schizophrenia. Because Ig-like domains are extracellular and mediate protein–protein interactions, they may represent accessible targets for autoantibody binding. Based on these observations, we hypothesized that other Ig superfamily members with similar domains may also be targeted by autoantibodies in patients with schizophrenia.

Among neural adhesion molecules belonging to the Ig superfamily, L1CAM represents a well-characterized synaptic adhesion molecule (Sytnyk et al., 2017). Similar to NCAM1, L1CAM plays a pivotal role in neurodevelopment and synapse formation (Duncan et al., 2021). L1CAM interacts with itself (homophilic interaction) and binds to Neuropilin-1 to regulate synaptic organization (Duncan et al., 2021). However, autoantibodies against L1CAM have not yet been identified. We therefore hypothesized that L1CAM autoantibodies may be present in patients with schizophrenia and may have clinical relevance, and tested this hypothesis in the present study.

Here, we report the identification of autoantibodies against L1CAM in a small subset of patients with schizophrenia (2 of 387). Moreover, IgG purified from the sera of patients positive for L1CAM autoantibodies was administered to mice to assess the resulting behavioral alterations.

## 2 Methods

### 2.1 Ethics

This study was performed in strict accordance with the Guidelines for Proper Conduct of Animal Experiments by the Science Council of Japan, the Helsinki Declaration, and the Ethical Guidelines for Medical and Health Research Involving Human Subjects in Japan. It was approved by the Committees on Gene Recombination Experiments, Human Ethics, and Animal Experiments of Institute of Science Tokyo (G2025-008A, M2000-1866, and A2024-099C2). All participants provided written informed consent.

### 2.2 Participants

Serum samples were obtained from 362 healthy controls (181 men and 181 women; ages 22–90 years; median, 49 years) and 387 patients with schizophrenia (195 men and 192 women; ages 16–84 years; median, 51 years). No significant differences in age were observed between the groups. Demographic data is shown in Table 1. The study cohort consisted of inpatients from the Institute of Science Tokyo Hospital, Kurita Hospital, and Takatsuki Hospital, as well as both inpatients and outpatients from the National Center of Neurology and Psychiatry Hospital, between April 1, 2016, and December 31, 2021. Schizophrenia was diagnosed according to the Fifth Edition of the Diagnostic and Statistical Manual of Mental Disorders (DSM-5) criteria, and the diagnosis was confirmed by agreement between at least two psychiatrists. Inclusion criteria for the patient group were a DSM-5 diagnosis of schizophrenia. Exclusion criteria included the presence of neurological disorders, such as autoimmune encephalitis or dementia, and a history of substance abuse. Healthy control sera were obtained from healthy volunteers and the BioBank at the Bioresource Research Center, Institute of Science Tokyo. Inclusion criteria for the control group were the absence of psychiatric disorders, and exclusion criteria included any history of psychiatric or neurological disorders, autoimmune diseases, malignancies, other severe medical conditions requiring hospitalization, and a history of substance abuse.

**Table 1:** **Demographic data**: Sex distribution was compared using the χ^2^ test. Age and disease duration are presented as median (IQR), and age was compared using the Mann–Whitney *U* test.

	Healthy control	Schizophrenia	*p* value
Number of participants	362	387	
Sex (female)	181 (50.0%)	192 (49.2)	0.91
Age (years)	49 [40–60]	51 [42–62]	0.47
disease duration	Not applicable	15 [7–24]	

### 2.3 Electroencephalography

Electroencephalography (EEG) was performed as part of routine clinical evaluation using standard scalp recordings. EEG findings were evaluated by a neurologist.

### 2.4 Serum samples and cerebrospinal fluid (CSF) samples

Serum samples were collected in the morning following an overnight fast or at least 2 h after food intake. In this study, serum samples were first screened for autoantibodies, primarily because serum collection is more feasible in patients with psychiatric disorders. For patients who tested positive, CSF analysis was subsequently performed when CSF collection was possible and after obtaining informed consent.

CSF analysis was included because CSF is considered to more directly reflect central nervous system immune activity and is widely used to support the clinical relevance of neuronal autoantibodies, particularly in the context of autoimmune encephalitis and autoimmune psychosis. Accordingly, both serum and CSF were analyzed in this study. As a result, CSF samples were obtained from one patient with schizophrenia who was positive for anti-L1CAM autoantibodies, whereas CSF was not available from the other patient.

CSF was collected in the morning within one month of serum collection. Serum and CSF samples were aliquoted and stored at −80 °C until use. Cell-based assays were performed using freshly thawed samples, avoiding repeated freeze–thaw cycles. CSF parameters, including total protein, albumin, glucose, IgG, and leukocyte counts, were analyzed by a certified clinical laboratory (SRL, Tokyo, Japan).

All procedures, including CSF collection, were approved by the Committees on Human Ethics of the Institute of Science Tokyo, as described in Section 2.1 (Ethics).

### 2.5 Cell culture and transfection

HeLa cells were cultured at 37 °C and 5% CO_2_ in Dulbecco's Modified Eagle Medium (Sigma, St. Louis, MO, USA), supplemented with 10% fetal bovine serum (FBS). The cells were transfected with plasmids using Lipofectamine 2000 (Thermo Fisher Scientific, Waltham, MA), according to the manufacturer's protocol. Mouse primary cortical neurons were prepared from embryonic day 15 (E15) C57BL/6J mouse embryos. The cerebral cortices were dissected and incubated with 0.05% trypsin in 4 mL of phosphate-buffered saline (PBS) at 37 °C for 15 min. The cells were then dissociated by pipetting. The cells were passed through a 70-μm cell strainer, collected by centrifugation, and cultured in Neurobasal Medium (Thermo Fisher Scientific) containing 2% B27, 0.5 mM L-glutamine, and 1% Penicillin/Streptomycin in the presence of 0.5 μM AraC. Immunocytochemistry of the primary neurons was performed at 14 days in vitro (DIV 14). These cultures predominantly consist of excitatory cortical neurons. as previously described (Brewer et al., 1993).

### 2.6 Construction of DNA vectors

**Fig. 1:**
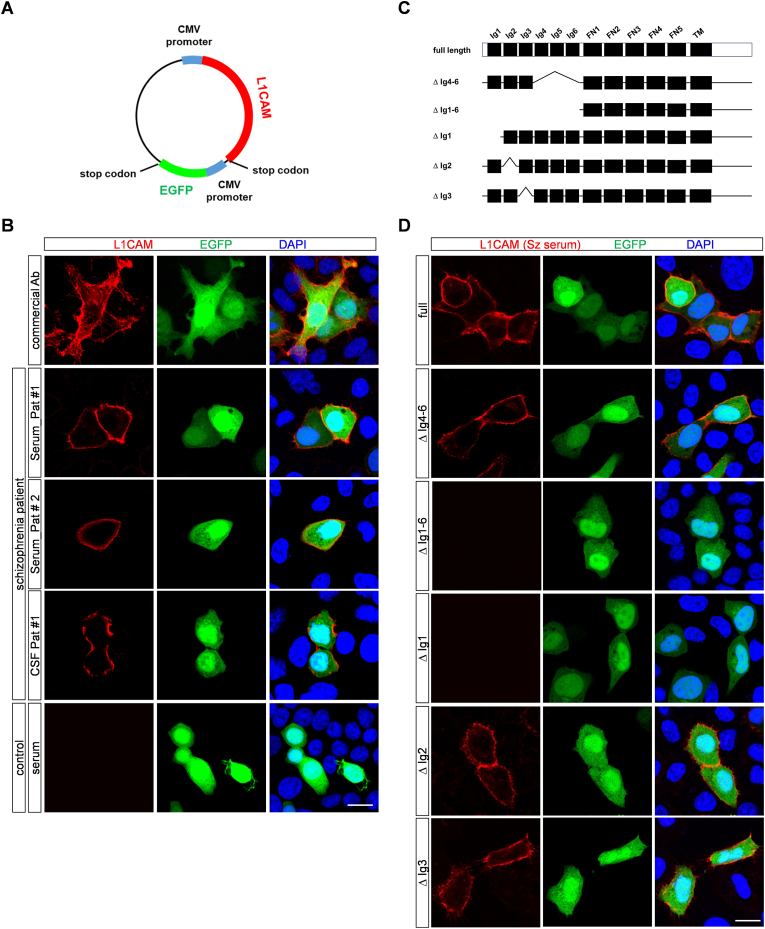
Identification of anti-L1CAM autoantibodies. A. Plasmid CMV-L1CAM-CMV-EGFP. B. Cell-based assay with immunocytochemistry using a commercial anti-L1CAM antibody. Sera and CSF obtained from patients with schizophrenia as well as sera from healthy controls were used. C. L1CAM deletion constructs. Ig: immunoglobulin domain, FN: Fibronectin type III domain, TM: transmembrane domain. D. Immunocytochemistry with HeLa cells transfected with L1CAM deletion constructs using serum from patient 1 with schizophrenia who tested positive for anti-L1CAM autoantibodies.

L1CAM and enhanced green fluorescent protein (EGFP) were cloned into a pRP vector (VectorBuilder), and expression was driven by the cytomegalovirus (CMV) promoter (Fig. 1A). L1CAM deletion constructs were generated using PrimeSTAR Max DNA Polymerase (Takara) and primers (Supplementary Table 1).

### 2.7 Cell-based assays, immunocytochemistry, and immunohistochemistry

A cell-based assay was performed in HeLa cells with an immunocytochemical approach to detect anti-L1CAM autoantibodies. HeLa cells were fixed using 2% paraformaldehyde (in phosphate-buffered saline [PBS]) at room temperature for 30 min and then treated with 0.1% Triton X-100 in PBS for 10 min. The cells were blocked for 30 min at room temperature using PBS with 10% FBS and then incubated with serum or a primary antibody diluted in a blocking buffer. Immunocytochemistry was performed with anti-L1CAM (1:200, MA5-11563, Thermo Fisher Scientific; 1:200, 67115-1-Ig, Proteintech).

For immunohistochemistry, brain samples were fixed with 4% paraformaldehyde and embedded in paraffin. Sagittal or coronal sections (5-μm thick) were cut using a microtome (Microm HM 335 E, GMI, Ramsey, USA). Sections were incubated with primary antibodies against L1CAM (1:200, 67115-1-Ig, Proteintech). To detect purified human IgG that had been injected into mice, brain sections were incubated directly with the secondary antibody without additional incubation with serum samples or purified human IgG.

Primary antibodies were detected using Cy3-conjugated anti-mouse IgG (1:500, 715-165-150, Jackson ImmunoResearch), whereas serum autoantibodies and purified human IgG injected into mice were detected using Cy3-conjugated anti-human IgG (1:500, 709-165-149, Jackson ImmunoResearch). Nuclei were stained with DAPI (0.2 μg/mL in PBS; DOJINDO). Images were acquired under an Olympus FV1200 confocal microscope (Tokyo, Japan).

Serum with an autoantibody titer of ≥1:30 were considered positive, as previously described (Katayama et al., 2025; Motokawa et al., 2025; Shiwaku et al., 2020, 2022, 2023; Steiner et al., 2013).

### 2.8 Mice

Male C57BL/6J mice were obtained from CLEA Japan (Tokyo, Japan) and housed in standard cages in a temperature- and humidity-controlled room under a 12-h light/dark cycle (lights on at 8:00 a.m.). A total of 40 male C57BL/6J mice were used in this study. Mice were group-housed (2 per cage) under standard laboratory conditions. Food and water were provided ad libitum. No additional environmental enrichment (e.g., running wheels) was used.

### 2.9 Behavioral tests

All behavioral tests were analyzed using a video-computerized tracking system (SMART, Panlab, Barcelona, Spain). All behavioral tests were performed using male C57BL/6J mice. IgG was administered at 8 weeks of age, and behavioral testing was performed at 9 weeks of age. The Y-maze test was conducted on day 1, followed by the open field test and the elevated plus maze test on day 2. A 3-h interval was introduced between the open field test and the elevated plus maze test. Investigators were blinded to group allocation during both experiments and data analysis.

#### 2.9.1 Open-field test

The open field test was conducted to evaluate locomotor activity and anxiety-like behavior (Seibenhener and Wooten, 2015). Mice were placed in an open-field box measuring 40 × 40 × 22 cm and allowed to explore freely for 10 min. The total distance traveled and the time spent in the central zone (20 × 20 cm) were measured.

#### 2.9.2 Elevated plus maze test

The elevated plus maze test was conducted to assess anxiety-like behavior (Walf and Frye, 2007). The elevated plus maze comprises two open arms and two closed arms (30 × 6 cm, with 15-cm walls, and is suspended 50 cm above the floor). Mice were placed in the central square of the maze and activity was recorded for 5 min. The time spent in the open and closed arms was measured.

#### 2.9.3 Y-maze test

The Y-maze test was conducted to assess cognitive function (Kraeuter et al., 2019). Mice were placed at the end of one arm and allowed to move freely through the maze for 8 min. The spontaneous alteration rate was calculated by dividing the number of entries into a new arm, different from the previous one, by the total number of transfers from one arm to another.

#### 2.9.4 Prepulse inhibition test

The test was conducted using sound-attenuating startle boxes (Panlab, Barcelona, Spain). After a 5-min acclimation period with background noise at 65 dB, each mouse was exposed to 10 blocks of six trial types presented in a pseudorandom order. The six trial types consisted of a startle-only trial, in which a 40-ms, 120-dB sound burst was presented, and five prepulse trials, in which a 20-ms prepulse stimulus (69, 73, 77, 81, or 85 dB) preceded the 120-dB startle stimulus by 100 ms. The maximum startle response was recorded for each startle stimulus.

### 2.10 Purification and intrathecal injection of IgG from serum

IgG was purified from the serum of patients with schizophrenia or a healthy control donor. The healthy control donor was negative for anti-L1CAM autoantibodies and was matched to the patient in terms of age and sex, with no history of psychiatric, neurological, or other systemic disorders. IgG was purified from serum using Protein G HP SpinTrap columns (28903134, Cytiva, USA) according to the manufacturer's protocol. Mice were anesthetized with 1% isoflurane using a small-animal anesthetizer (TK-7, BioMachinery, Japan). Using a micropipette puller (model P-1000, Sutter Instrument, USA) and FemtoJet (Eppendorf, NY, USA), 3 μg of purified IgG in 2 μL of solution was injected into the subarachnoid space of the frontal cortex of 8-week-old mice at a speed of 1 μL/min. Assuming a mouse CSF volume of 40 μL (Pardridge, 2016), the dilution rate used for purification and injection corresponded to a 100-fold dilution of the serum. Because the antibody titer of anti-L1CAM autoantibodies in serum is 1:300, we considered that the dilution rate used was appropriate for autoantibody analysis. This procedure was performed as previously described (Shiwaku et al., 2022, 2023).

### 2.11 Statistical analysis

Statistical analysis was performed using GraphPad Prism 8.4.3 (GraphPad Software, Inc., CA, USA). Data groups were first analyzed by one-way ANOVA, followed by Tukey's honestly significant difference (HSD) test for multiple comparisons, unless otherwise noted in the figure legends. Categorical variables were compared using the χ^2^ test, and continuous variables were compared using the Mann–Whitney *U* test. Statistical significance was set at a p-value <0.05. The exact value of n, the definition of center and dispersion, and precision measures are described in the figure legends.

## 3 Results

Using a cell-based assay with L1CAM-expressing HeLa cells, we identified anti-L1CAM autoantibodies in the serum of 2 of 387 patients with schizophrenia (Fig. 1A and B). These autoantibodies were also detected in the CSF of one of the antibody-positive patients, whereas CSF samples were not available for the other patient (Fig. 1B–Table 2). In contrast, anti-L1CAM autoantibodies were not detected in healthy control sera (0/362; Fig. 1B). Statistical comparison using Fisher's exact test showed no significant difference between patients with schizophrenia and healthy controls (2/387 vs. 0/362, *p* = 0.50). Consistent with these findings, patient sera showed immunoreactivity against primary neuronal cultures derived from mice, consistent with binding to L1CAM expressed on neuronal surfaces (Supplementary Fig. 1).

**Table 2:** Clinical characteristics of anti-L1CAM autoantibody–positive patients.

Case no./sex/age	Illness duration (years)	Antibody titer (CBA) (serum/CSF)	EEG	Neuroimaging	BACS Z-score, Comorbidity
1/F/48	3	300/3	generalized slowing (basic rhythm 7 Hz)	MRI, mild cortical atrophy	−3.07
2/F/52	30	300/NA	NA	MRI, mild nonspecific T2 prolongation in the cerebral cortex	−2.94 diabetes mellitus

EEG, electroencephalogram; F, female; M, male; MRI, magnetic resonance imaging; CSF, cerebrospinal fluid; CBA, cell based assay; NA, not available; BACS, Brief Assessment of Cognition in Schizophrenia.

Cell-based assays showed that sera from anti-L1CAM autoantibody–positive patients did not exhibit immunoreactivity against other synaptic molecules, including NCAM1, NLGN1–4, ephrin B1–B3, ERBB4, NRG1, NR1, NR2, and GABA_A_Rα1 (data not shown).

The clinical characteristics of the two patients with anti-L1CAM autoantibodies identified by cell-based assay are summarized in Table 2. Both patients presented with hallucinations and delusions for more than one year, and exhibited significant cognitive deficits based on their BACS scores. Patient 1 showed the following scores: Verbal Memory, 36; Digit Sequencing, 11; Token Motor Task, 46; Verbal Fluency, 16; Symbol Coding, 31; and Tower of London, 18, with a composite BACS Z-score of −3.07. Patient 2 showed the following scores: Verbal Memory, 31; Digit Sequencing, 18; Token Motor Task, 50; Verbal Fluency, 17; Symbol Coding, 34; and Tower of London, 13, with a composite BACS Z-score of −2.94. Previous studies have reported that patients with schizophrenia typically show BACS composite scores around −1 to −2, with scores below approximately −1.35 considered indicative of cognitive impairment. In this context, the observed scores (−3.07 and −2.94) suggest substantial cognitive impairment relative to typical schizophrenia cohorts. For Patient 1, the PANSS total score was 80 (positive, 20; negative, 18; general psychopathology, 42), whereas PANSS data were not available for Patient 2. Patient 1 showed mild generalized slowing on electroencephalography (EEG), characterized by a dominant background rhythm of approximately 7 Hz (Table 2). Further clinical evaluation, including CSF analysis, was performed in patient 1. CSF analysis in patient 1 revealed an elevated total protein level of 67 mg/dL (reference range: 15–45 mg/dL) and an albumin level of 232 mg/L (reference range: <200 mg/L). CSF IgG was also elevated at 12.6 mg/dL (reference range: 0–4 mg/dL), whereas IgA (1.0 mg/dL) and IgM (<0.2 mg/dL) were within the normal range. The IgG index was within the normal range (0.53), suggesting the absence of intrathecal IgG synthesis. The elevated CSF total protein, albumin, and IgG levels may therefore be consistent with blood–CSF barrier dysfunction. Glucose (47 mg/dL), chloride (>120 mEq/L), and lactate dehydrogenase (13 U/L) were within normal limits. The CSF cell count was normal (3 cells/μL), with 100% mononuclear cells. These findings indicate no evidence of pleocytosis. Aside from the presence of anti-L1CAM autoantibodies, no marked inflammatory abnormalities were observed in routine blood tests. Patient 2 had a history of diabetes mellitus, whereas neither patient had any other notable medical history. In particular, no autoimmune diseases or malignancies were identified in either patient.

To identify the epitope recognized by anti-L1CAM autoantibodies, we performed domain-mapping analysis using L1CAM deletion constructs (Fig. 1C). L1CAM consists of six Ig domains, five FNIII domains, and a transmembrane region. This analysis revealed that the autoantibodies specifically targeted the Ig1 domain (Fig. 1D).

**Fig. 2:**
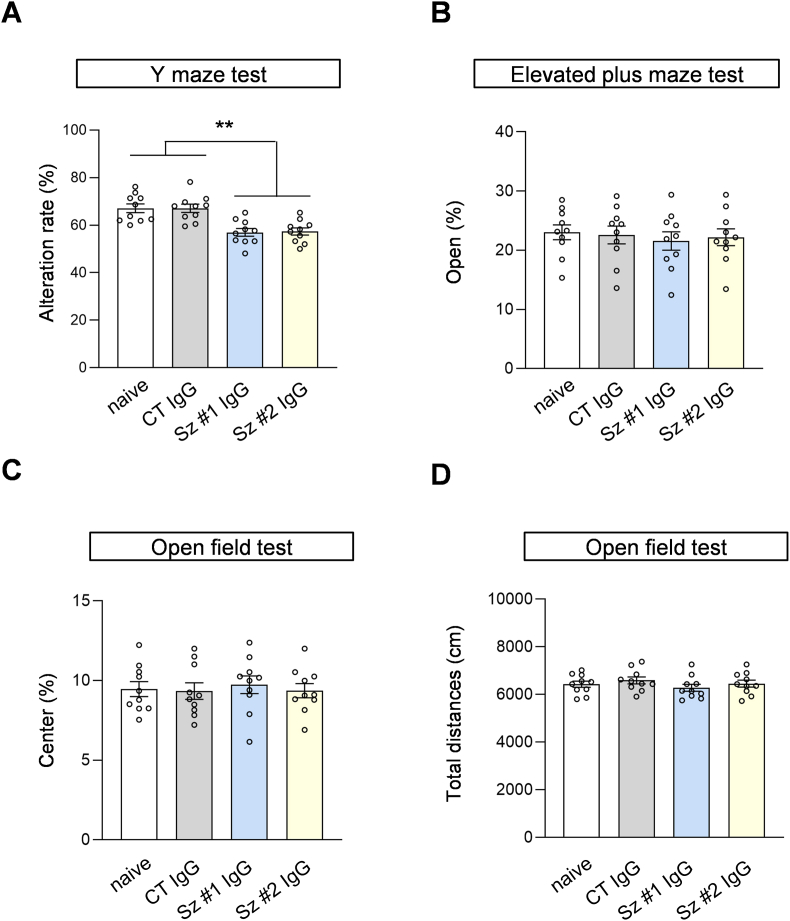
Anti-L1CAM autoantibodies induce cognitive impairments in mice A. Alteration ratios in the Y-maze test after the administration of purified IgG obtained from patients with schizophrenia or from a healthy control. **p < 0.01 (N = 10 mice per group; one-way ANOVA followed by Tukey's HSD test). B. Time in the open arm regions in the elevated plus maze test. There was no significant difference between the groups. (N = 10 mice for each group; one-way ANOVA) C. Time in the center region in the open field test. There was no significant difference between the groups. (N = 10 mice for each group; one-way ANOVA). D. Total distances in the open field test. There was no significant difference between the groups. (N = 10 mice for each group; one-way ANOVA).

To examine the behavioral effects of anti-L1CAM autoantibodies, IgG derived from antibody-positive patients was administered into the CSF of mice. IgG derived from Patients 1 and 2 with schizophrenia who were positive for anti-L1CAM autoantibodies remained detectable in the mouse brain one week after intrathecal administration (Supplementary Fig. 2). Mice receiving patient-derived IgG showed impaired cognitive performance in the Y-maze test (Fig. 2A). One-way ANOVA revealed a significant group effect (F(3,36) = 11.17, p = 3.55 × 10^−5^, η^2^ = 0.48), and post hoc analysis using Tukey's HSD test showed significant differences between control groups (naive and healthy control) and patient groups (Patient 1 and Patient 2) (naive vs Patient 1, p = 0.0011; naive vs Patient 2, p = 0.0010; healthy control vs Patient 1, p = 0.0023; healthy control vs Patient 2, p = 0.0020). In contrast, no significant differences were observed in locomotor activity or anxiety-like behavior in the open field test, anxiety-like behavior in the elevated plus maze test, or sensorimotor gating, as assessed by the prepulse inhibition test. (Fig. 2B–D, Supplementary Fig. 3; one-way ANOVA: open field center time, F(3,36) = 0.084, p = 0.968, η^2^ = 0.0078; total distance traveled, F(3,36) = 0.412, p = 0.745, η^2^ = 0.033; elevated plus maze open arm time, F(3,36) = 0.593, p = 0.623, η^2^ = 0.047; for the prepulse inhibition test, see Supplementary Fig. 3 legend).

## 4 Discussion

In this study, we identified autoantibodies against L1CAM in a small subset of patients with schizophrenia (2 of 387), which target the Ig1 domain of L1CAM. Intracerebroventricular administration of IgG purified from these patients induced cognitive impairment in mice.

L1CAM plays a key role in synapse formation and brain development. Impaired L1CAM function causes intellectual disability in humans (Fransen et al., 1997; Schäfer and Altevogt, 2010; Wong et al., 1995), and knockout mice lacking L1cam exhibit cognitive deficits (Dahme et al., 1997; Fransen et al., 1998). In this study, we demonstrated that IgG from patients positive for anti-L1CAM autoantibodies induced cognitive impairment in mice; however, the underlying mechanisms remain to be elucidated. Anti-L1CAM autoantibodies may interfere directly with L1CAM function or indirectly affect microglial activity, thereby influencing neuroinflammation and synaptic pruning (Shiwaku, 2025). Furthermore, autoantibodies against NCAM1 and NRXN1 have been reported to disrupt intermolecular interactions, and anti-L1CAM autoantibodies may similarly impair L1CAM homophilic interactions, potentially leading to L1CAM dysfunction (Shiwaku et al., 2022, 2023). Further elucidation of these mechanisms will be an important subject for future studies.

Schizophrenia is increasingly recognized as a syndrome of heterogeneous and incompletely understood etiological origins (Owen et al., 2016). Within this framework, neuronal autoantibodies have been proposed as potential markers that may help define biologically meaningful subgroups of psychosis (Pollak et al., 2020). For example, autoimmune psychosis represents a subgroup characterized by the presence of pathogenic autoantibodies, including those targeting NMDA and GABA receptors (Lennox et al., 2017). In addition, synaptic autoantibodies against molecules such as NCAM1 and NRXN1 have been identified in patients with schizophrenia (Shiwaku et al., 2022, 2023). Although anti-L1CAM autoantibodies were identified in only a small number of patients in the present study, their detection may further expand the spectrum of autoantibody-associated subgroups within psychotic disorders. These findings raise the possibility that L1CAM autoantibodies may represent a hypothesis-generating marker within the emerging framework of autoimmune psychosis. Future studies will be required to determine the prevalence and clinical relevance of L1CAM autoantibodies across psychotic disorders, including autoimmune psychosis and schizophrenia.

Regarding cognitive function, the observation of EEG slow waves in an anti-L1CAM autoantibody–positive patient with schizophrenia is of interest. Similar EEG abnormalities have been reported in schizophrenia patients with anti-NCAM1 or anti-GABA receptor autoantibodies, suggesting that such patterns may, in some cases, have an autoantibody-related basis (Shiwaku et al., 2020, 2022). Further investigation is warranted to determine whether patients with schizophrenia exhibiting EEG slowing also possess neuronal autoantibodies. As multiple neuronal autoantibodies are known to cause autoimmune encephalitis, future studies could explore the potential spectrum of L1CAM autoantibody–associated encephalitis, encephalopathy, and schizophrenia, including cases presenting with EEG slowing.

The Ig1 domain is also the epitope targeted by NCAM1 autoantibodies (Shiwaku et al., 2022). However, the presence of L1CAM and NCAM1 autoantibodies was mutually exclusive in our cohort: patients positive for one were negative for the other. Despite the structural similarities among the Ig domains, their amino acid sequences differ (Bork et al., 1994; Wasserman and Fickett, 1998), suggesting that anti-L1CAM autoantibodies recognize specific sequence motifs rather than shared structural features.

This study has several limitations. First, the prevalence of anti-L1CAM autoantibodies in schizophrenia is low, suggesting that they are unlikely to contribute to the majority of schizophrenia cases. Nevertheless, rare findings can provide important biological insights. For example, rare genetic variants with high odds ratios for schizophrenia are often present in only a small fraction of patients, yet have substantially advanced our understanding of disease mechanisms. In this context, the presence of anti-L1CAM autoantibodies (approximately 0.5%, 2/387 patients) may provide insight into the immunological aspects of schizophrenia (Farsi et al., 2023; Mukai et al., 2019; Nagahama et al., 2020; Singh et al., 2022; Toyoda et al., 2025). Second, although IgG purified from anti-L1CAM autoantibody–positive patients induced cognitive impairment in mice, we cannot completely exclude the possibility that other unidentified factors, including additional autoantibodies, may have contributed to the observed effects. While the tested autoantibodies, including those against NMDA receptors, GABA receptors, NCAM1, and NRXN1, were negative, thyroid-related autoantibodies, including anti–NH2-terminal of α-enolase (NAE) antibodies, as well as other uncharacterized autoantibodies, were not assessed in this study. Further studies will be required to confirm the specificity of the observed effects. In addition, because the present study was conducted only in male mice, potential sex differences in the pathogenic effects of L1CAM antibodies remain to be elucidated. Third, because only two patients were positive for anti-L1CAM autoantibodies, detailed characterization of the associated clinical features was limited. Although cognitive impairment was observed in these patients, larger studies will be necessary to determine the consistency and clinical relevance of this finding. Furthermore, because behavioral analyses in the present study primarily focused on cognitive domains, the relationship between L1CAM autoantibodies and other core symptoms of schizophrenia remains to be established. Future studies incorporating larger cohorts and comprehensive phenotypic and treatment-response analyses will be required to clarify the role of L1CAM autoantibodies, as well as to delineate similarities and differences between L1CAM and other neuronal autoantibodies, including NCAM1, in schizophrenia. Finally, given the low prevalence and limited number of positive cases, the present findings should be considered exploratory and hypothesis-generating. Future studies with larger cohorts and mechanistic investigations will be required to validate these observations and determine whether these autoantibodies are causally involved in disease processes.

In summary, we identified anti-L1CAM autoantibodies in two patients with schizophrenia. Further studies with larger cohorts will be required to clarify their clinical significance and potential role in disease mechanisms.

## CRediT authorship contribution statement

**Shingo Katayama:** Data curation, Formal analysis, Investigation, Methodology, Validation, Writing – review & editing. **Yukiko Motokawa:** Data curation, Formal analysis, Investigation, Methodology, Software, Validation, Visualization, Writing – review & editing. **Gayatri Nayanar:** Formal analysis, Investigation, Methodology, Validation, Writing – review & editing. **Saori Toyoda:** Data curation, Formal analysis, Investigation, Methodology, Validation, Writing – review & editing. **Hiroaki Hori:** Resources, Supervision, Validation, Writing – review & editing. **Yohsuke Yagi:** Resources, Supervision, Validation, Writing – review & editing. **Sayuri Ishiwata:** Data curation, Formal analysis, Investigation, Resources, Validation, Writing – review & editing. **Kinya Ishikawa:** Resources, Supervision, Validation, Writing – review & editing. **Hiroshi Kunugi:** Resources, Supervision, Validation, Writing – review & editing. **Hidehiko Takahashi:** Funding acquisition, Resources, Supervision, Validation, Writing – review & editing. **Hiroki Shiwaku:** Conceptualization, Data curation, Formal analysis, Funding acquisition, Investigation, Methodology, Project administration, Resources, Software, Supervision, Validation, Visualization, Writing – original draft, Writing – review & editing.

## Declaration of competing interest

The authors declare that they have no known competing financial interests or personal relationships that could have appeared to influence the work reported in this paper.

## Data Availability

Data will be made available on request.
