# Supplementary material for: Identification of autoantibodies against L1CAM in patients with schizophrenia
Source: Brain Behav Immun Health. 2026 Jun 15;55:101288. doi: 10.1016/j.bbih.2026.101288 (PMC13293718; doi:10.1016/j.bbih.2026.101288)
Supplement: Multimedia component 1 [file mmc1.pdf]

Supplemental information

Identification of autoantibodies against L1CAM in patients with schizophrenia

Shingo Katayama[1, #], Yukiko Motokawa[1, #], Gayatri Nayanar[1], Saori Toyoda[1], Hiroaki Hori[2], Yohsuke Yagi[3], Sayuri Ishiwata[2], Kinya Ishikawa[4], Hiroshi Kunugi[2, 5], Hidehiko Takahashi[1], Hiroki Shiwaku[1, *]

1. Department of Psychiatry and Behavioral Sciences, Institute of Science Tokyo, Yushima, Bunkyo-ku, Tokyo 113-8510, Japan.
2. Department of Behavioral Medicine, National Institute of Mental Health, National Center of Neurology and Psychiatry, Tokyo 187-8553, Japan
3. Department of Neurology and Neurological Science, Institute of Science Tokyo, Tokyo, Japan.
4. Department of Personalized Genomic Medicine for Health, Institute of Science Tokyo, Tokyo, Japan.
5. Department of Psychiatry, Teikyo University School of Medicine, Tokyo 173-8605, Japan

Supplementary Figure 1

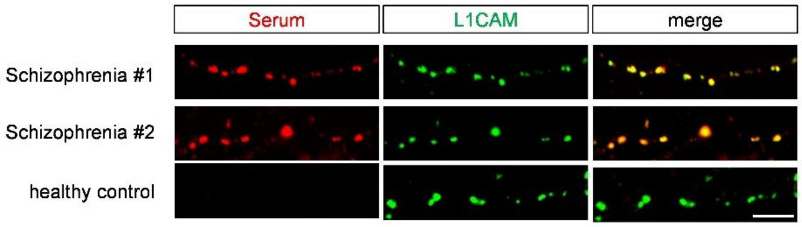

Supplementary Figure 1. Anti-L1CAM autoantibodies from schizophrenia patients reacted with L1CAM expressed by primary cultured neurons. Immunocytochemical analysis of primary neurons from mice using a commercial anti-L1CAM antibody, and IgGs from schizophrenia patients, and IgGs from a healthy control. Bar: 5 μm.

Supplementary Figure 2

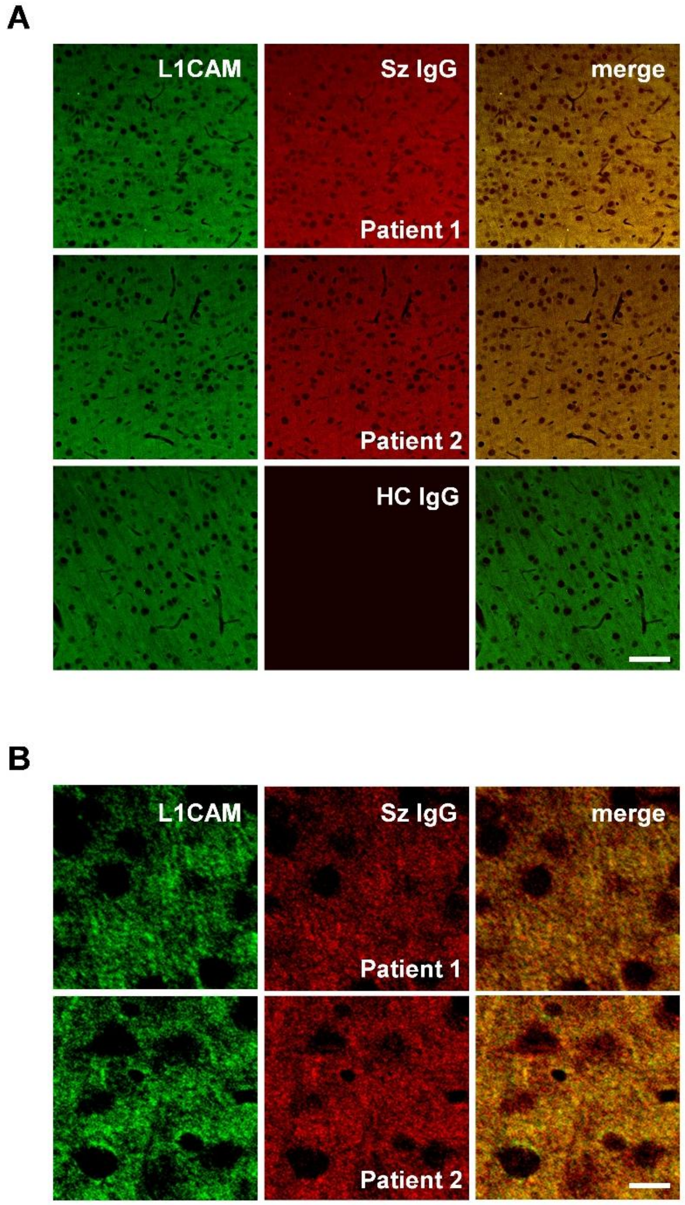

Supplementary Figure 2. Detection of intrathecally administered IgG with anti-L1CAM autoantibodies.

A. IgG purified from anti-L1CAM autoantibody–positive Patients 1 and 2 with schizophrenia was intrathecally injected into 8-week-old mice. Injected human IgG remained detectable in the brain at 9 weeks of age, whereas IgG purified from healthy controls was not detected. Scale bar, 100 µm.

B. Higher magnification of A. Scale bar, 5 µm.

Supplementary Figure 3

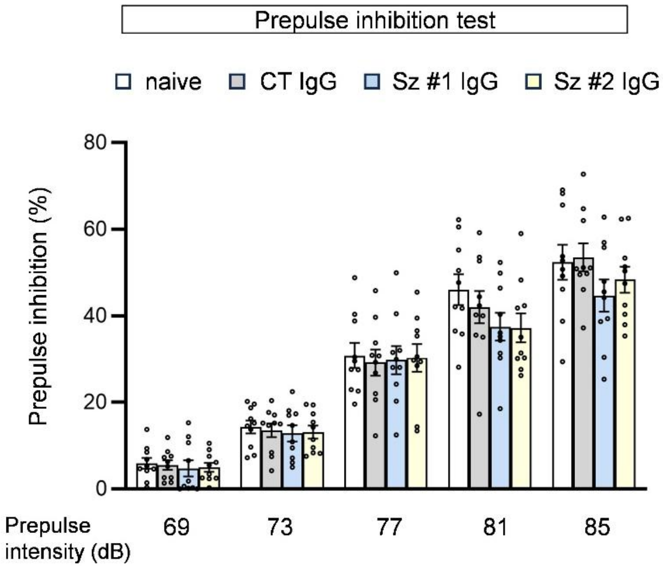

Supplementary Figure 3. Prepulse inhibition rates in mice injected with IgG purified from a healthy control or anti-L1CAM autoantibody–positive patients with schizophrenia.

There was no significant difference among the groups. N = 10 mice per group. One-way ANOVA: 69 dB, $F(3, 36) = 0.1434$, $p = 0.9332$, $\eta^2 = 0.0118$; 73 dB, $F(3, 36) = 0.1566$, $p = 0.9247$, $\eta^2 = 0.0129$; 77 dB, $F(3, 36) = 0.0472$, $p = 0.9862$, $\eta^2 = 0.0039$; 81 dB, $F(3, 36) = 1.4715$, $p = 0.2386$, $\eta^2 = 0.1092$; 85 dB, $F(3, 36) = 1.2936$, $p = 0.2914$, $\eta^2 = 0.0973$.

**Oligonucleotides**
L1CAM ΔIg1-6 primer forward: 5'-ACGTGTGGACCGAACTGGATGTGGTG-3'
L1CAM Δ Ig1-6 primer reverse: 5'-GTTCGGTCCACACGTACCGCAGCGC-3'
L1CAM ΔIg4-6 primer forward: 5'-ATGTCACCCTCTTGGTGGTGGGGAGC-3'
L1CAM ΔIg4-6 primer reverse: 5'-CCAAGAGGGTGACATAGTACGCATG-3'
L1CAM ΔIg1 primer forward: 5'-TGATGGAGCCAAGTGCAGAGCCTCTC-3'
L1CAM ΔIg1 primer reverse: 5'-CACTTGGCTCCATCACATGGTGTCC-3'
L1CAM ΔIg2 primer forward: 5'-ATGAGATCAGCATGATTGACAGGAAG-3'
L1CAM ΔIg2 primer reverse: 5'-TCATGCTGATCTCATGGGACATGGC-3'
L1CAM ΔIg3 primer forward: 5'-ACATCTGCCAGAGCCATCTATATGGG-3'
L1CAM ΔIg3 primer reverse: 5'-GGCTCTGGCAGATGTAGTCTGAGTG-3'

Supplementary Table 1. Oligonucleotides used in this study.
